# Evaluating Intercropping Indices in Grass–Clover Mixtures and Their Impact on Maize Silage Yield

**DOI:** 10.3390/plants15020293

**Published:** 2026-01-18

**Authors:** Marko Zupanič, Miran Podvršnik, Vilma Sem, Boštjan Kristan, Ludvik Rihter, Tomaž Žnidaršič, Branko Kramberger

**Affiliations:** 1 Faculty of Agriculture and Life Sciences, University of Maribor, Pivola 10, 2311 Hoče, Slovenia; 2 Agricultural and Forestry Institute Maribor, Vinarska ulica 14, 2000 Maribor, Slovenia; 3 Agricultural Institute of Slovenia, Hacquetova ulica 17, 1000 Ljubljana, Slovenia

**Keywords:** clover, competition indices, grass, intercropping, maize, management practices, mixed cropping

## Abstract

A field experiment was conducted in 2019–2020 and 2020–2021 at Rogoza, Fala, and Brežice in Slovenia to examine the biological viability of a mixed intercropping system and the effect of winter catch crops (WCCs) on maize growth parameters. The experiment included Italian ryegrass (IR) in pure stands, fertilized with nitrogen (N) in spring (70 kg N ha^−1^), mixtures of crimson clover and red clover 50:50 (C), and intercropping between IR and C (IR+C). Neither mixture was fertilized with N in spring. We evaluated different competition indices and biological efficiency. Relative crowding coefficient (RCC) and actual yield loss (AYL) exceeded 1, indicating a benefit of IR+C intercropping. The IR in intercropping was more aggressive, as indicated by positive aggressivity (A) and a competitive ratio (CR) > 1, and it dominated over C in IR+C (that had negative A values and CR < 1). The competitive balance index (Cb) differed from zero, the relative yield total (RYT) was 2.24, the land equivalent coefficient (LEC) exceeded 0.25, the area–time equivalent ratio (ATER) exceeded 1, and land use efficiency (LUE) exceeded 100%. IR+C exhibited the highest total aboveground dry matter yield of maize (29.22 t ha^−1^), the highest nitrogen content in dry matter grain yield of maize (206.35 kg ha^−1^), the highest nitrogen and potassium content in maize stover (105.7 and 105.7 kg ha^−1^, respectively), and the highest nitrogen and potassium content in the total aboveground dry matter of maize (312 and 267.3 kg ha^−1^, respectively). The C/N ratio in dry matter yield of IR was 45.35, and in IR+C it was 33.43, which means that the mixture had a positive effect on nutrient release in maize. The ryegrass–clover mixture, according to the calculated biological indices, had advantages over pure stands and had a positive effect on maize yield.

## 1. Introduction

Climate change has become a major challenge to agriculture, freshwater resources, and the food security of billions of people [1]. There have been clear shifts in global surface temperature, precipitation, evaporation, and the frequency of extreme events [2]. In addition, the world population is projected to exceed 9 billion by 2050 [3,4]; this population growth will amplify pressure on natural resources, underscoring the need for their sustainable management, particularly in agricultural production systems [5].

Climatic anomalies increasingly threaten crop production and are closely associated with ongoing global warming [6,7]. As agriculture is highly sensitive to weather variability, changes in abiotic factors, such as temperature and precipitation, can substantially affect crop growth and yield [8,9]. Climate change influences crop quantity and quality both through direct and indirect effects, prompting adjustments in agronomic management practices, including nutrient management, irrigation, plant protection, cropping system design, and the selection of climate-resilient crops and varieties [10,11,12,13,14]. In the context of the critical task of supplying safe, high-quality food to an expanding global population [4], intercropping systems provide a practical solution for achieving higher yields, meeting food demand while supporting sustainable agriculture [15,16,17].

Intercropping is an eco-friendly system wherein two or more species are grown together on the same land for part or all of the growing season [18,19,20,21,22,23]. By producing a faster, denser canopy, this system can achieve the same output on a small cultivated area while reducing soil erosion [24]. Moreover, intercropping can reduce the incidence of diseases, pests, and weed damage and enhance crop competitiveness against weeds [25,26,27,28,29]. These advantages are attributed to complementary and more efficient capture of water, light, temperature-related microclimates, nutrients, and atmospheric resources, which increase yields, improve land productivity, and enhance biodiversity and ecosystem services [30,31,32,33,34]. Considering the decline in available land resources and the rapid population growth, intercropping is crucial for meeting rising global food demand and improving yield and profitability per unit area [35]. This approach often includes legumes, which contribute to improved soil quality, reduced climate-related risks and crop failure, enhanced biodiversity, and increased resource use efficiency [36].

Successful intercropping depends on the careful selection of companion crops, plant spacing, sowing time, and maturity, with complementary resource use enabling mixtures to outperform monocultures [37,38,39]. For example, Grasses (*Poaceae*) and legumes (*Fabaceae*) are often intercropped because they complement one another in livestock diets and N acquisition. Legumes establish symbiotic relationships with atmospheric N-fixing bacteria [40,41,42,43]. As a result of N fixation by symbiotic rhizobacteria in legume root nodules [44], the herbage production of grass–legume mixtures under moderate N fertilizer levels can match that of the best pure stands of non-legumes receiving high inputs of N fertilizer [45], providing both environmental and economic benefits for farmers [46]. Beyond N dynamics, grasses and legumes differ in root morphology and spatial distribution, supporting distinct soil microbial communities [47,48]. These differences decrease competition for water and mineral nutrients in mixtures compared to pure crops stand, increase soil biotic diversity, and promote facilitative interactions that improve soil resource exploitation [49,50]. Root niche differentiation refers to the ability of coexisting species to exploit different soil layers, thereby reducing direct belowground competition and enabling complementary acquisition of water and nutrients [51,52]. In grass–legume mixtures, perennial ryegrass (*Lolium perenne* L.) typically develops a shallow, fibrous root system with a large proportion of the root biomass concentrated in the upper soil layers (e.g., the upper 10 cm), whereas red clover (*Trifolium pratense* L.) maintains a persistent taproot that can access deeper subsoil resources [53,54]. This vertical partitioning of rooting space provides a mechanistic basis for expecting complementarity [54]. Despite structural differences between grass and legume roots, intercropping can reshape the root distribution and architecture by favoring different root types and can modify rhizosphere exudation patterns [55,56].

Intercropping performance is evaluated using various indices. For biological evaluation, these include the relative yield total (RYT) [57,58], land equivalent coefficient (LEC) [59], area–time equivalent ratio (ATER) [60], land-use efficiency (LUE) [18,60,61], system productivity index (SPI) [62], and percentage yield difference (PYD) [63]. For competition, the commonly used indices include the relative crowding coefficient (RCC) [64,65], aggressivity (A) [66,67], competitive ratio (CR) [24,68], competitive balance index (Cb) [69], and actual yield loss (AYL) [70].

Winter catch crops (WCCs) can positively affect the subsequent crop in the arable rotation by increasing water-holding capacity [71,72,73], soil porosity, and soil aggregate stability [71,72]. WCCs can also reduce erosion [74,75,76,77,78,79], promote atmospheric N fixation [73,80,81,82], increase soil organic matter [83], decrease weed pressure [71,84,85], and enhance soil nutrient availability for the following crops [82,86]. Moreover, they can reduce nitrate-leaching risk [87,88,89] while maintaining maize yield [90]. Therefore, adopting WCCs is both environmentally and economically justified in maize cultivation [91].

To the best of our knowledge, no study has explicitly evaluated the biological efficiency of an Italian ryegrass (IR) and clover intercropping system (IR+C), and the intensity of competition among these components within such a system remains unclear. Therefore, using multiple indices, in the present study, we aimed to evaluate the biological efficiency of an IR+C system, assess the competitive intensity between the component crops, and determine the effects of intercropping of WCCs on maize yield. We hypothesized that the IR+C system would be more stable and result in higher maize yield compared to IR and C.

## 2. Results

All presented data represent the averages of 2 years (2019–2020 and 2020–2021) and 3 locations in Slovenia (Rogoza, Fala, and Brežice). Our results revealed that the dry matter yield (DMY) of IR and clovers in pure stands was higher than that of both crops in the intercropping system (Table 1).

All total RYT values, calculated based on both DMY and N, were greater than 1 (Table 2). While the total RYT on a N basis was 0.20 lower than that on a DMY basis, it still exceeded 1. In both cases, the LEC exceeded 0.25.

The ATER calculated on both a DMY and a N basis was greater than 1, the LUE exceeded 100%, the PYD was below 100%, and Cb was greater than 0 in both cases. Moreover, all calculated AYL values were positive; however, the total AYL calculated on a DMY basis was 0.41 higher than that calculated on a N basis. Similarly, the SPI calculated from DMY was 62.5-fold higher than that calculated based on N. The competitive ratio (CR) of IR, calculated on both DMY and N bases, was higher than that of C. In addition, the aggressivity values calculate based on both a DMY and a N basis for IR within the intercropping systems (IR+C) were positive, whereas those for C were negative. The total relative crowding coefficient (RCC), calculated on both DMY and N bases, was greater than 1. The land saved (LSAV), calculated using DMY, was 4.37% higher than that calculated using N (Table 2).

Table 3 presents correlations between the parameters of IR and C within the IR+C systems. A single degree symbol (°) indicates that the parameter was calculated based on dry matter yield (DMY), whereas a double degree symbol (°°) indicates calculation based on N content in DMY (N in DMY). The amount of N in the DMY of IR (DMY_IR_) in the intercropping system was significantly (*p* < 0.05) negatively correlated with the DMY of C (DMY_C_), CR of C (CR_C_ °°), and aggressivity of C (A_C_ °°). The CR of IR (CR_IR_ °) was also negatively correlated with DMY_C_ in the intercropping system, RYT of C (RYT_C_ °), RYT_C_ °°, RCC of C (RCC_C_ °), RCC_C_ °°, CR_C_ °, A_C_ °, AYL of C (AYL_C_ °), and AYL_C_ °°. The RYT (RYT_IR_ °), RCC (RCC_IR_ °), aggressivity of Italian ryegrass (A_IR_ °), and AYL of IR (AYL_IR_ °) were significantly (*p* < 0.05) negatively associated with RYT_C_ °, RCC_C_ °, AYL_C_ °, CR_C_ °°, and A_C_ °° in the IR+C intercropping system. Moreover, DMY_IR_, RYT_IR_ °°, RCC_IR_ °°, CR_IR_ °°, A_IR_ °°, and AYL_IR_ °° were also significantly (*p* < 0.05) negatively associated with CR_C_ °° and A_C_ °° in intercropping system. Furthermore, other correlations such as those between DMY_IR_ and N_C_, and between DMY_IR_ and A_C_ °, approached the level of significance (*p* < 0.1).

The DMY of the whole aboveground biomass of maize was higher in the IR+C treatment (29.22 t ha^−1^) than in the IR and C treatment (28.32 and 26.35 t ha^−1^, respectively; Table 4). The potassium content in dry matter grain yield of maize (KDMY) and phosphorus content in dry matter grain yield of maize (PDMY) in IR+C (60.6 and 44.8 kg ha^−1^, respectively) were comparable to those in IR (64.9 and 47.9 kg ha^−1^, respectively). The potassium content in maize stover (KMS) and total potassium content in the whole aboveground dry matter of maize (TKC) in IR+C (206.7 and 267.3 kg ha^−1^) were higher than that in IR (163.9 and 228.8 kg ha^−1^, respectively) and C (149.7 and 204.5 kg ha^−1^, respectively). No significant differences (*p* > 0.05) were observed for dry matter grain yield of maize (GYM), NDMY, NMS, PMS, TNC, and TPC (Table 4).

## 3. Discussion

Forage grasses generally have a higher forage production capacity than forage legumes. Among these, IR often achieves significantly elevated DMY, likely owing to its strong photosynthetic capacity [92]. However, the biological performance of an IR+C intercropping system is unclear. In the present study, we conducted a field experiment over two growing seasons (2019–2020 and 2020–2021) at three locations in Slovenia (Rogoza, Fala, and Brežice) to investigate the biological performance of an IR+C intercropping system, intensity of competition among the WCCs in the mixture IR+C, and effects of WCCs on maize yield. We demonstrated indicated that pure IR stands perform comparably to an IR+C intercrop but yield more than pure crimson clover stands, which is consistent with previous findings [93,94]. In our experiment, pure IR stands produced more green forage and higher DMY than the IR component within the intercropping system (Table 1), likely owing to the decreased seeding rates of individual species in mixtures and competition effects from the companion crop [70]. Nevertheless, when assessed at the whole-system level (IR+C), integrating C into IR increased the total DMY relative to the corresponding pure stands. Similarly, Kumar et al. [95] reported lower yields of IR and red clover in pure stands than in mixtures. Simić et al. [96] also reported higher total yields of both IR and red clover in mixtures than in pure stands, although their pure stands achieved higher yields than those observed in our study (Table 1).

A total RYT greater than 1 generally indicates a yield advantage for forage mixtures, with grass–legume intercropping often outperforming the corresponding monocultures [97,98]. This advantage often occurs in grass-clover combinations because the crops can exploit environmental and land resources more efficiently [99,100]. In our study, the RYT for the intercropping systems was greater than 1, indicating that mixing C with IR enhances the growth and yield of the companion species. This pattern indicates that interspecific facilitation outweighs interspecific competition, resulting in an elevated LUE [67,101], which is consistent with previously reported RYT values [102]. However, in a 3-year experiment conducted by Dirks et al. [54], the mixture of white clover (*Trifolium repens* L.) and perennial ryegrass (*Lolium perenne* L.) achieved lower RYT values than those observed in our experiment (Table 2).

In addition, the LEC in our intercropping system was greater than 0.25 (Table 2), indicating an advantage of intercropping over pure stands [59]. The ATER values were greater than 1, demonstrating that intercropping improved the efficiency of crop use area within a single growing season [103]. Notably, this reflects the same advantage suggested by RYT values, whereby IR+C mixtures outperformed corresponding monocultures.

Plant competition within intercropping systems is a key determinant of growth and yield [65]. Intercropping is the most advantageous when interspecific competition is lower than intraspecific competition and mutualistic interactions occur between component species [104,105]. In an intercropping system, each species has its own RCC value [64,65]. In the present study, our analysis of the RCC revealed decreased interspecific competition in the intercropping system compared to intraspecific competition in pure crop stands. Moreover, IR consistently exhibited higher RCC values than the companion C, indicating its dominance within the mixture. In contrast, C exhibited RCC values lower than 1 (Table 2), suggesting a yield disadvantage [24,64], whereas the RCC values of IR were greater than 1 (Table 2), indicating a yield advantage [70]. Overall, these results suggest that intercropping conferred a yield advantage, as the product RCC_IR_ × RCC_C_ exceeded 1 [105], demonstrating that the positive interactions between the two species outweighed competitive effects. Moreover, the difference between the CR of IR and C was positive, indicating that IR dominated over clovers. The CR_IR_ exceeded 1, implying that IR exerted a negative (suppressive) effect on the companion clovers [24,68,106]. Consistently, the CR of IR was considerably higher than that of C (Table 2), confirming its stronger competitive ability in forage mixtures [68]. Overall, these results indicate that intraspecific competition exerted a stronger influence on competitive outcomes than interspecific competition in our study.

In the present study, the positive and higher values for partial AYL_IR_ for IR demonstrated that IR dominated over C, which exhibited lower partial AYL_C_ values (Table 2). This result indicates a yield gain that is likely attributed to the positive effect of C on IR when grown in combination [70,99,102,107]. The partial AYL values for IR exhibited a clear yield benefit for the crop when combined with C [102], and the positive A_IR_ value of 0.77 (Table 2) suggests the dominance of IR over C [108]. Moreover, DMY-based land saving-calculation (LSAV) was 4.37% higher than that based on N, with 54.32% and 49.95% of land being saved by IR+C intercropping, respectively. These results suggest that this intercropping technique may be applied to improve land-use efficiency in other agricultural systems.

Moreover, in terms of the correlations between the parameters of IR and C within the intercropping mixture, all correlations (R) were negative. This indicates that an increase in each IR parameter (e.g., DMY, N in DM) was associated with a decrease in the corresponding C parameter within the mixture. This result also suggests a pronounced competitive relationship between the two crop species, in which the superior performance of IR is disadvantageous to the clovers. Moreover, some correlations approached the threshold of significance (*p* < 0.1). Overall, these results support the conclusion that IR does not strongly influences C.

In the present study, N fertilizer was applied to IR in spring. IR responds strongly to applied N and utilizes it more efficiently than C. Red clover (*Trifolium pratense* L.) is a widely grown forage legume that forms a nitrogen-fixing symbiosis with strains of *Rhizobium leguminosarum* symbiovar *trifolii* [109,110]. Symbiotically fixed N was also available to IR, enhancing its competitive ability for light and nutrients. As grass exploits soil N more efficiently than C, it grows faster and taller, overtopping the clovers and reducing both its proportion and DMY contribution in the mixture [111]. Increased N availability also promotes enhanced development of belowground grass biomass [112,113].

Compared to our results, Kramberger et al. [114] reported lower total aboveground maize biomass and maize grain yields for IR in pure stands, crimson clover (CRC) in pure stands, and a 50:50 IR+CRC mixture. Moreover, in their IR pure stands, the N content in maize grain and the total aboveground biomass of maize were lower than those observed in our experiment (Table 4). However, in contrast to our experiment, Kramberger et al. [114] did not fertilize IR with N, and the aboveground part of IR was plowed into the soil. In the CRC pure stand, the authors reported higher N content in maize grain and in total aboveground maize biomass than in the mixture. In our experiment, the IR+C (50:50) mixture resulted in higher N content in maize grain and total aboveground maize biomass than pure stands (Table 4). These results indicate that, in the absence of initial N fertilization, IR grown in pure stands or mixtures with a high proportion of IR resulted in decreased total aboveground maize biomass and grain yield. In contrast, mixtures with a high proportion of C enhanced maize yield and N uptake in the harvested yield. Consistently, Gollner et al. [115] reported that maize yields after pure stands of IR or mixtures dominated by grass were lower than those achieved by legume-rich mixtures. In our experiment, the C/N ratio in the DMY of IR was higher (45.35) than that in IR+C (33.43), indicating that the IR+C biomass degraded earlier, making nutrients available to maize sooner. Thus, the positive effect of the WCCs on maize yield could have been more pronounced in our experiment if the entire aboveground catch crop biomass, rather than only the belowground portion, had been incorporated into the soil.

## 4. Materials and Methods

### 4.1. Experimental Site, Treatments, and Crop Management

Field experiments were conducted across two winter-summer seasons (2019–2020 and 2020–2021) at three locations in Slovenia: Rogoza (46°29′59.15″ N, 15°40′49.75″ E; 266 m asl.), Fala (46°32′43.35″ N, 15°27′15.67″ E; 306 m asl.), and Brežice (45°54′24.17″ N, 15°35′30.00″ E; 162 m asl.). Three winter catch crops (WCCs) were evaluated: Italian ryegrass (*Lolium multiflorum* L., cultivar ‘Melquatro’), crimson clover (*Trifolium incarnatum* L., cultivar ‘Heusers ostsaat’), and red clover (*Trifolium pratense* L., cultivar ‘Global’). A randomized complete block design was implemented at all sites. Sand, silt, and clay contents were determined using the sieving sedimentation method [116] and soil texture was classified using the texture triangle [117]. The soils were classified as clay at Rogoza and silty clay at Brežice. Soil organic matter content was the highest in Brežice (2.2%) and lowest in Fala (1.7%) in the first year, and again the highest in Brežice (2.8%) and lowest in Rogoza (1.5%) in the second year. Seedbeds were prepared to a depth of 8 cm using a power harrow. Before sowing the winter catch crops (WCCs), fields received 50 kg N, 70 kg P_2_O_5_, and 120 kg K_2_O ha^−1^. The WCCs were sown in late August and harvested the following May. In spring, only the pure stand of Italian ryegrass was top-dressed with 70 kg N ha^−1^ as potassium ammonium nitrate (27% N). Sowing and harvest dates varied among site-years due to differences in the harvest timing of the preceding cash-crop and site-specific rainfall conditions. After WCCs harvest, 130 kg N, 110 kg P_2_O_5_, and 180 kg K_2_O ha^−1^ were applied; fields were then plowed and prepared for maize using pre-sowing tillage. Operational details and weather by site year are summarized in Table 5.

Six random soil samples per plot were collected at two depths (0–30 and 31–60 cm) at three times: late November, early May, and early October after maize harvest. Plant available P_2_O_5_ and K_2_O were measured using the AL method [118]. Soil organic matter was determined by the Walkley–Black method [119]. Total plant N was calculated from whole-plant biomass using the Kjeldahl method [120].

To determine the DMY of WCCs, six samples of aboveground biomass per treatment were collected using 0.25 m^2^ quadrats and clipped with electric shears at a 5 cm stubble height. Samples were oven-dried at 60 °C for 48 h and weighed. Each block comprised three treatments, and each treatment plot measured 3000 m^2^. In the IR+C treatments, after sampling, the aboveground biomass of IR and C was separated, and each sample was individually weighed. Samples were then oven-dried at 60 °C for 48 h and reweighed. After sampling, the WCCs were mown, and the biomass was ensiled. Before sowing maize, only the belowground part was incorporated into the soil. To determine maize DMY and grain yield, ten plants per treatment were harvested by cutting 10 cm above the ground with shears, then oven dried at 60 °C for 48 h.

The details of maize seeding for all sites, including field operations and weather conditions, are summarized in Table 5.

To determine maize DMY and grain yield, 10 plants per treatment were harvested by cutting them 10 cm above the ground with shears, before oven-drying them at 60 °C for 48 h. The N content was calculated from whole-aboveground plant biomass using the Kjeldahl method [121,122]. The P content in maize grain and maize stover samples was determined using atomic absorption spectrometry according to ISO 6869:2000 [123], and that in the maize grain and maize stover samples was determined using an ultraviolet-visible spectrophotometer [124] according to ISO 6491:1998 [125]. The pH of the soil samples was determined according to ISO 10390 [126].

### 4.2. Evaluation of the Biological Performance of the Grass–Legume Intercropping System

#### 4.2.1. Calculation of Relative Yield Total (RYT)

The RYT quantifies the proportion of monocropped area required to achieve the yield obtained by intercropping [57,58,127]. The RYT for the total DMY was calculated as described in Equations (1)–(3):

RYT = RYT_IR_ + RYT_C_ (1)

(2) $${\mathrm{RYT}}_{\mathrm{IR}}=\frac{IDMIR}{SDMIR\times ZIR}$$

(3) $${\mathrm{RYT}}_{C}=\frac{IDMC}{SDMC\times ZC}$$

where RYT_IR_ and RYT_C_ are partial RYT for IR and C, IDMIR and IDMC are the intercrop DMY for IR and C, and SDMIR and SDMC represent the DMY of pure stands of IR and C, and ZIR and ZC denote the sown proportions of IR and C in the mixture, respectively.

An RYT value greater than 1 indicates a yield advantage for intercropping [128,129], whereas values below 1 indicate that intercropping reduced yield compared to the corresponding pure stand [129,130,131]. An RYT of 1.0 indicates that intercropping produces the same yields as a pure stand [132]. In this study, the nutritional relationships between intercrop components were evaluated using the RYT [56,133,134,135]. The RYT for N was computed similarly to that of RYT for the total DMY, wherein N uptake was used in the equation instead of DMY. The land equivalent ratio [136] is mathematically equivalent to the RYT and is used to compare biomass production in mixtures of forage species with that of the corresponding pure stands [57].

#### 4.2.2. Calculation of Land Equivalent Coefficient (LEC)

The LEC is calculated as the product of RYT_IR_ and RYT_C_ using Equation (4), as previously described by Adetiloye et al. [59]. When the minimum productivity is 25%, an LEC value exceeding 0.25 indicates a crop advantage. The LEC reflects the strength of the interaction between component crops in an intercropping system. In a two-crop mixture, LEC values greater than 0.25 are considered advantageous.

(4) $$\mathrm{LEC}=\frac{IDMIR}{SDMIR}\times \frac{IDMC}{SDMC}$$

The LEC for N was calculated similarly to that for the total DMY, in which N uptake was used instead of the DMY in the equation.

#### 4.2.3. Calculation of System Productivity Index (SPI)

To evaluate the productivity and stability of the intercropping systems, the SPI [62], which converts clover yield (secondary crop) into IR equivalents (the primary crop), was calculated as described by Agegnehu et al. [137] and Machiani et al. [67] (Equation (5)).

(5) $$\mathrm{SPI}=IDMIR+\left(\frac{SDMIR}{SDMC}\times IDMC\right)$$

The SPI for N was computed similarly to that for total DMY, based on the corresponding N uptake.

#### 4.2.4. Calculation of Area Time Equivalent Ratio (ATER)

Mead and Willey [60] proposed the ATER as a modification of RYT, incorporating crop duration. In this study, the ATER was used to compare the yield advantage of intercropping IR with C compared to monocropping, accounting for the time each component occupied the field from planting to harvest [137,138] (Equation (6)):

(6) $$\mathrm{ATER}\;=\;\frac{\left(\frac{IDMIR}{SDMIR}\times dir\right)+\left(\frac{IDMC}{SDMC}\times dc\right)}{T}$$

where dir is the growth period (days) between planting and maturity for IR, dc is the growth period (days) between planting and maturity for clovers, and T is the growth period in days for the intercropping system.

The ATER for N was computed similarly to that for total DMY, replacing DMY with N uptake in the formula.

#### 4.2.5. Calculation of Percentage Yield Difference (PYD)

The PYD quantifies the percent difference in yield between a pure stand and its corresponding intercrop [63], whereby the yield of pure stand is set at 100%, and any reduction in the yield of one component is typically compensated by an increase in the companion crop. Unlike most other indices, a larger PYD indicates lower intercropping efficiency, whereas a smaller or negative PYD indicates higher efficiency [63].

(7) $$\mathrm{PYD}=100-\left(\frac{SDMIR-IDMIR}{SDMIR}+\frac{SDMC-IDMC}{SDMC}\right)\;\times \;100$$

The PYD for N was similarly computed, with the DMY replaced by N uptake in the corresponding equation.

#### 4.2.6. Calculation of Land Use Efficiency (LUE)

The LUE was calculated using Equation (8) as previously described [18,60,61].

(8) $$\mathrm{LUE}=\left(RYTs+\frac{ATER}{2}\right)\times 100$$

The LUE for N was computed similarly, with N uptake replacing the DMY in the corresponding equation.

### 4.3. Competition Indices

#### 4.3.1. Calculation of Competitive Ratio (CR)

The CR, introduced by Rao and Willey [24] and Dhima et al. [68], was used to assess the competitive ability of the component crops in the intercropping system [139,140] and calculated using Equations (9) and (10).

(9) $${\mathrm{CR}}_{\mathrm{IR}}=\frac{\frac{IDMIR}{SDMIR}}{\frac{IDMC}{SDMC}}\times \frac{ZC}{ZIR}$$

where CR_IR_ is the competitive ratio of IR, ZC is the sown ratio of C in the mixture, and ZIR is the sown ratio of IR in the mixture. The CR for N was computed similarly to that for total DMY, wherein N uptake of corresponding nutrients, instead of DMY, was used.

(10) $${\mathrm{CR}}_{C}=\frac{\frac{IDMC}{SDMC}}{\frac{IDMIR}{SDMIR}}\times \frac{ZIR}{ZC}$$

where CR_C_ is the competitive ratio of clovers.

A CR_IR_ value below 1 indicates a positive benefit of intercropping, suggesting that IR can be grown in association with clover, whereas a CR_C_ greater than 1 indicates negative benefit. If the difference between CR_IR_ and CR_C_ is 0, then IR and C exhibit equal competitive ability [141,142]. If, by subtracting CR_C_ from CR_IR,_ a positive value is estimated, then intercropped IR is dominant. In contrast, a negative value indicates that the companion clover dominates IR. The CR for the N was computed similarly to that of CR for total DMY, wherein N uptake replaced the DMY in the equation.

The CR for the N was computed similarly to that of CR for total DMY, wherein N uptake replaced the DMY in the equation.

#### 4.3.2. Calculation of Aggressivity (A)

The A index was used as a competition index to quantify the extent to which the relative yield of one crop in the mixture exceeded that of the other, as defined in Equations (11) and (12) [66,67].

(11) $$A_{\mathrm{IR}}\;=\frac{IDMIR}{SDMIR\times ZIR}-\frac{IDMC}{SDMC\times ZC}$$

(12) $$A_{C}=\frac{IDMC}{SDMC\times ZC}-\frac{IDMIR}{SDMIR\times ZIR}$$

where A_IR_ is the aggressivity of IR and A_C_ is the aggressivity of clovers. The A for N was computed similarly to that for total dry matter, wherein N uptake was used instead of dry matter in the equation. If A_IR_ or A_C_ equals 0, both crops in the intercropping system are equally competitive. A positive A_IR_ denotes dominance of IR over C, whereas a negative value indicates that C is the dominating species [108].

The A for the N was computed similarly to that of A for total DMY, wherein N uptake replaced the DMY in the equation.

#### 4.3.3. Calculation of Relative Crowding Coefficient (RCC)

The RCC was used as a competitive power index to evaluate the relative dominance or aggressiveness of IR over C, and vice versa, within the intercropping system [64,65,143] using Equations (13)–(15).

RCC = RCC_IR_ × RCC_C_ (13)

(14) $${\mathrm{RCC}}_{\mathrm{IR}}=\frac{IDMIR\times ZC}{(SDMIR-IDMIR)\times ZIR}$$

(15) $${\mathrm{RCC}}_{C}=\frac{IDMC\times ZIR}{(SDMC-IDMC)\times ZC}$$

where RCC_IR_ is the RCC of IR, and RCC_C_ is that of C. An RCC value greater than indicates a benefit of intercropping. In contrast, an RCC value less than 1 indicates a disadvantage, and a value of 1 denotes neither advantage nor disadvantage of the intercropping system [108,138].

The RCC for N was computed similarly to that of RCC for total DMY, wherein N uptake replaced DMY in the formula.

#### 4.3.4. Calculation of Actual Yield Loss (AYL)

The AYL was used to characterize competition between intercrop components, as it reflects the equivalent yield gain or loss of each crop relative to its respective pure stand [99]. Unlike the RYT, the AYL explicitly accounts for the actual sown proportions of land occupied by the component crops in the field. Positive and negative AYL values indicate an advantage or disadvantage of intercropping, respectively, when yields are compared on a per-plant basis [70]. Banik et al. [70] emphasized that the AYL index offers precise insights into both interspecific and intraspecific competition within an intercropping system, as well as the behavior of the component crops. AYL values greater than 0 indicate that the intercropping has an advantage over pure stands, whereas AYL values below 0 indicate that the pure stands outperform the intercrop. Positive or negative AYL_IR__ and AYL_C_ values reflect the respective contributions of IR and clover to the mixture, indicating a gain or a loss in the overall system [144,145]. The AYL was calculated using Equations (16)–(18):

AYL = AYL_IR_ + AYL_C_ (16)

(17) $${\mathrm{AYL}}_{\mathrm{IR}}=(\frac{IDMIR}{SDMIR}\times \frac{100}{ZIR})-1$$

(18) $${\mathrm{AYL}}_{C}=(\frac{IDMC}{SDMC}\times \frac{100}{ZC})-1$$

The AYL for the N was computed similarly to that of AYL for total DMY, wherein N uptake replaced DMY in the formula.

#### 4.3.5. Calculation of Percentage of Land Saved (%LSAV)

The percentage of LSAV was calculated according to Equation (19) [146], as follows:

(19) $$\%\mathrm{LS}\;=\;100\;-\;\left(\frac{1}{RYT}\;\times \;100\right)$$

The percentage of LSAV for the N was computed similarly to that for total DMY, wherein N uptake replaced DMY in the formula.

#### 4.3.6. Calculation of Competitive Balance Index (Cb)

The Cb of one species relative to the other in the intercropping system was calculated using Equation (20), as previously described [69].

(20) $$\mathrm{Cb}=\log[\left(\frac{IDMIR}{SDMIR}\right):\left(\frac{IDMC}{SDMC}\right)]$$

where the symbols are the same as those used for the RYT. A Cb value of 0 indicates no competition or equal competitive abilities [65], whereas any other value indicates that one species is less affected or has more advantage in the intercropping system than the other.

The Cb for the N was computed similarly to that of Cb for total DMY, wherein N uptake replaced DMY in the formula.

### 4.4. Statistical Analyses

Linear mixed-effects models (LMERs) were fitted to test the effects of WCC treatments (fixed effects) on DMYM, GYM, NDMY, KDMY, PDMY, NMS, KMS, PMS, TNC, TKC, and TPC. Year and location were included as random effects to account for variance associated with measurements from the same year or site. As residual diagnostics exhibited no deviations from homoscedasticity or normality, no data transformations were applied. Pairwise differences among treatments were assessed using Fisher’s least significant difference test. Analyses were conducted using R version 4.2.2 [147] (R Foundation for Statistical Computing, Vienna, Austria). LMERs were fitted with the *lmer* function in the lme4 package [148], *p*-values for fixed effects were obtained via Satterthwaite approximations in lmerTest [149], and multiple comparisons were performed with *glht* [150]. To estimate the correlation between the indices of IR (DMYIR, NIR, RYTIR, RCCIR, CRIR, AIR, AYLIR) and C (DMYC, NC, RYTC, RCCC, CRC, AC, and AYLC), the Spearman correlation coefficient was used.

## 5. Conclusions

In this study, we evaluated the biological viability of mixed intercropping and the effect of WCCs on maize growth parameters We demonstrated that:

1. The dry matter yield of IR in pure stands and C was significantly higher (*p* < 0.05) compared with their dry matter yield under intercropping, whereas the total yield of the IR+C mixture was higher than that of each WCCs grown in a pure stand.
2. Analysis of competition indices confirmed intercropping advantages of IR+C, with RCC, AYL, RYT, and ATER values all greater than 1, LUE greater than 100%, and LEC exceeding 0.25.
3. Moreover, IR in the IR+C mixture was more aggressive (A > 0; CR > 1) and dominated C (negative A; CR < 1), with a Cb value different than 0, indicating unbalanced competition.
4. The total maize DMY was the highest in the IR+C (29.22 t ha^−1^) compared to IR and C.
5. Furthermore, intercropping of IR+C without spring N fertilization achieved maize grain yield, N content in maize grain yield, P content in maize stover, and total phosphorus content in the whole aboveground dry matter of maize comparable to those obtained with spring N fertilization of IR, with no significant differences (*p* > 0.05).

Collectively, these results indicate that while intercropping of IR+C enables maize yield to be managed without spring N fertilization of WCCs, it alters K dynamics in maize, highlighting the need for closer K (and partly P) management in maize. This study supports sustainable maize production with reduced mineral N inputs, while acknowledging the potential for increased competition for nutrients. The IR+C intercropping without spring N achieves yields and nutrient outputs in maize comparable to those following N-fertilized pure IR, while improving several biological efficiency indices.

Further research should focus on identifying catch crop species or mixtures that most consistently maximize subsequent maize yield, as well as determining the optimal proportion of each catch crop component within mixtures. These findings should be validated across multiple sites and years and supported by measurements of biomass production and soil nitrogen dynamics.

## Figures and Tables

**Table 1:** Dry matter yield (DMY) of pure stands and Italian ryegrass–clover intercropping system.

Treatment	DMY (t ha^−1^)	
	Grass	Clover
IR	5.06 ^a^	-
C	-	4.03 ^a^
IR+C	3.79 ^b^	1.48 ^b^

^a,b^ Means followed by different superscript letters in the same column indicate significant differences between treatments (*p* < 0.05); IR, Italian ryegrass; C, mixture of crimson clover and red clover (50:50).

**Table 2:** Biological and competition indices of Italian ryegrass–clovers (IR+C) intercropping systems.

Treatment	RYT ° (C)	RYT ° (IR)	RYT ° (Total)	RYT °° (C)	RYT °° (IR)	RYT °° (Total)	LEC °	LEC °°
IR+C	0.73	1.51	2.24	0.77	1.26	2.04	1.06	0.94
	**ATER °**	**ATER °°**	**LUE °** **(%)**	**LUE °°** **(%)**	**PYD °** **(%)**	**PYD °°** **(%)**	**Cb °**	**Cb °°**
	2.24	2.04	337.38	306.34	12.46	2.11	0.71	0.47
	**SPI °** **(kg ha^−1^)**	**SPI °°** **(kg ha^−1^)**	**AYL °** **(C)**	**AYL °** **(IR)**	**AYL °** **(total)**	**AYL °°** **(C)**	**AYL °°** **(IR)**	**AYL °°** **(total)**
	5658.52	90.52	0.46	2.02	2.49	0.55	1.52	2.08
	**CR °** **(C)**	**CR °** **(IR)**	**CR °°** **(C)**	**CR °°** **(IR)**	**A °** **(C)**	**A °** **(IR)**	**A °°** **(C)**	**A °°** **(IR)**
	0.55	2.28	0.69	1.79	**−0.77**	**0.77**	**−0.48**	**0.48**
	**RCC °** **(C)**	**RCC °** **(IR)**	**RCC °** **(total)**	**RCC °°** **(C)**	**RCC °°** **(IR)**	**RCC °°** **(total)**	**LSAV °** **(%)**	**LSAV °°** **(%)**
	0.62	6.56	3.07	0.69	7.40	3.65	54.32	49.95

**°** calculated based on DMY; **°°** calculated based on N content in DMY. DMY, dry matter yield; IR, Italian ryegrass; C, mixture of crimson clover and red clover (50:50); RYT, relative yield total; LEC, land equivalent coefficient; ATER, area–time equivalent ratio; LUE, land-use efficiency; PYD, percentage yield difference; Cb, competitive balance index; SPI, system productivity index; AYL, actual yield loss; CR, competitive ratio; A, aggressivity; RCC, relative crowding coefficient; LSAV, land saved.

**Table 3:** Correlations values between parameters of Italian ryegrass (IR) and clovers (C) in the IR+C intercropping system.

	Italian Ryegrass											
Clover	DMY_IR_	N_IR_	RYT_IR_ °	RYT_IR_ °°	RCC_IR_ °	RCC_IR_ °°	CR_IR_ °	CR_IR_ °°	A_IR_ °	A_IR_ °°	AYL_IR_ °	AYL_IR_ °°
DMY_C_	−0.60	−0.89 **	−0.77	−0.60	−0.66	−0.60	−0.89 **	−0.66	−0.77	−0.66	−0.77	−0.60
N_C_	−0.83 *	−0.77	−0.66	−0.71	−0.77	−0.71	−0.77	−0.77	−0.66	−0.77	−0.66	−0.71
RYT_C_ °	−0.66	−0.77	−0.83 *	−0.54	−0.77	−0.54	−0.94 **	−0.60	−0.83 *	−0.60	−0.83 *	−0.54
RYT_C_ °°	−0.77	−0.83 *	−0.94 **	−0.60	−0.89 **	−0.60	−1.00 **	−0.71	−0.94 **	−0.71	−0.94 **	−0.60
RCC_C_ °	−0.66	−0.77	−0.83 *	−0.54	−0.77	−0.54	−0.94 **	−0.60	−0.83 *	−0.60	−0.83 *	−0.54
RCC_C_ °°	−0.77	−0.83 *	−0.94 **	−0.60	−0.89 **	−0.60	−1.00 **	−0.71	−0.94 **	−0.71	−0.94 **	−0.60
CR_C_ °	−0.77	−0.83 *	−0.94 **	−0.60	−0.89 **	−0.60	−1.00 **	−0.71	−0.94 **	−0.71	−0.94 **	−0.60
CR_C_ °°	−0.94 **	−0.89 **	−0.77	−0.94 **	−0.83 *	−0.94 **	−0.71	−1.00 **	−0.77	−1.00 **	−0.77	−0.94 **
A_C_ °	−0.83 *	−0.77	−1.00 **	−0.71	−0.94 **	−0.71	−0.94 **	−0.77	−1.00 **	−0.77	−1.00 **	−0.71
A_C_ °°	−0.94 **	−0.89 **	−0.77	−0.94 **	−0.83 *	−0.94 **	−0.71	−1.00 **	−0.77	−1.00 **	−0.77	−0.94 **
AYL_C_ °	−0.66	−0.77	−0.83 *	−0.54	−0.77	−0.54	−0.94 **	−0.60	−0.83 *	−0.60	−0.83 *	−0.54
AYL_C_ °°	−0.77	−0.83 *	−0.94 **	−0.60	−0.89 **	−0.60	−1.00 **	−0.71	−0.94 **	−0.71	−0.94 **	−0.60

Significance levels are indicated as: * *p* < 0.10; ** *p* < 0.05; **°** calculated based on DMY; **°°** calculated based on N content in DMY; DMY_IR_, dry matter yield of Italian ryegrass; N_IR_, nitrogen content in DMY of Italian ryegrass; RYT_IR_, relative yield total of Italian ryegrass; RCC_IR_, relative crowding coefficient of Italian ryegrass; CR_IR_, competitive ratio of Italian ryegrass; A_IR_, aggressivity of Italian ryegrass; AYL_IR_, actual yield loss of Italian ryegrass. DMY_C_, dry matter yield of clovers; N_C_, nitrogen content in DMY of clovers; RYT_C_, relative yield total of clovers; RCC_C_, relative crowding coefficient of clovers; CR_C_, competitive ratio of clovers; A_C_, aggressivity of clovers; AYL_C_, actual yield loss of clovers.

**Table 4:** Effects of treatments on different parameters in maize.

Parameter	Treatment		
	IR	C	IR+C
DMYM (t ha^−1^)	28.32 ^ab^	26.35 ^b^	29.22 ^a^
GYM (t ha^−1^)	15.98	14.89	15.95
NDMY (kg ha^−1^)	204.34	190.15	206.35
KDMY (kg ha^−1^)	64.9 ^a^	54.8 ^b^	60.6 ^ab^
PDMY (kg ha^−1^)	47.9 ^a^	40 ^b^	44.8 ^ab^
NMS (kg ha^−1^)	94.4	89.7	105.7
KMS (kg ha^−1^)	163.9 ^ab^	149.7 ^b^	206.7 ^a^
PMS (kg ha^−1^)	21.6	14	21.3
TNC (kg ha^−1^)	298.7	279.9	312
TKC (kg ha^−1^)	228.8 ^ab^	204.5 ^b^	267.3 ^a^
TPC (kg ha^−1^)	69.6	53.9	66.1

^a,b^ Means followed by different superscript letters in the same row indicate significant differences between treatments (*p* < 0.05). DMYM, dry matter yield of the whole aboveground biomass of maize; GYM, dry matter grain yield of maize; NDMY, nitrogen content in dry matter grain yield of maize; KDMY, potassium content in dry matter grain yield of maize; PDMY, phosphorus content in dry matter grain yield of maize; NMS, nitrogen content in maize stover; KMS, potassium content in maize stover; PMS, phosphorus content in maize stover; TNC, total nitrogen content in the whole aboveground dry matter of maize; TKC, total potassium content in the whole aboveground dry matter of maize; TPC, total phosphorus content in the whole aboveground dry matter of maize; IR, Italian ryegrass; C, mixture of crimson clover and red clover (50:50).

**Table 5:** Site descriptions, field operations, and prevailing weather conditions for the 2020 and 2021 winter catch crops and maize growing seasons at three sites.

Site Characteristics	2019–2020			2020–2021		
	Rogoza	Fala	Brežice	Rogoza	Fala	Brežice
Sand (%)	33.8	53.4	23.5	31.2	28.2	21.4
Silt (%)	47.5	30.4	55.8	42.4	45.6	57.5
Clay (%)	18.7	16.2	20.7	26.4	25.6	21.1
Soil texture	clay	sandy clay	silty clay	clay	clay	silty clay
Soil organic matter (%)	1.8	1.7	2.2	1.5	2.2	2.8
Soil pH (CaCl_2_)	6.2	6.3	5.3	6.5	6.4	5.8
P_2_O_5_ (mg/100 g soil)	16.1	14.7	9.2	13.0	15.2	10.2
K_2_O (mg/100 g soil)	20.6	16.9	20.2	18.7	14.5	19.4
Previous crop of WCCs	oilseed rape	barley	barley	barley	wheat	wheat
Sowing date of WCCs	27 August	28 August	29 August	26 August	28 August	29 August
Fertilizer before sowing WCCs (50 kg N; 70 kg P_2_O_5_; 120 kg K_2_O ha^−1^)	the entire experimental area			the entire experimental area		
Nitrogen application of WCCs in spring (kg N ha^−1^)	70 *	70 *	70 *	70 *	70 *	70 *
Harvesting date of WCCs	6 May	3 May	2 May	10 May	8 May	9 May
Plot size (m^2^)	3000	3000	3000	3000	3000	3000
Sum of precipitation during the growth period of WCCs (from the end of August to the beginning of May) (mm)	470	498	648	562	569	680
Plowed and seedbed preparation	7 May	4 May	4 May	11 May	9 May	10 May
Fertilizer application before sowing maize (130 kg N; 110 kg P_2_O_5_; 180 kg K_2_O ha^−1^)	Entire experimental area			Entire experimental area		
Sowing date of maize	8 May	5 May	5 May	12 May	10 May	11 May
Maize plant density was 8 plants per m^−2^	Entire experimental area			Entire experimental area		
Application of an herbicide after maize emergence (Adengo, 0.44 L ha^−1^)	Entire experimental area			Entire experimental area		
Fertilization and cultivation of maize in June (kg N ha^−1^)	60	60	60	60	60	60
Sum of precipitation during the growth period of maize (from the beginning of May to the beginning of October) (mm)	648.8	617.7	680.6	523.4	502.2	535.2
Harvesting date of maize	18 October	20 October	22 October	21 October	23 October	24 October

* At all three locations, only the Italian ryegrass treatment in a pure stand involved fertilization with N. WCCs, winter catch crops.

## Data Availability

The original contributions presented in this study are included in the article. Further inquiries can be directed to the corresponding author.

## Abbreviations

The following abbreviations are used in this manuscript.

A	Aggressivity
ATER	Area–time equivalent ratio
AYL	Actual yield loss
C	Mixtures of crimson clover and red clover 50:50
Cb	Competitive balance index
CR	Competitive ratio
DMY	Dry matter yield
DMYM	Dry matter yield of the whole aboveground biomass of maize
GYM	Dry matter grain yield of maize
IR	Italian ryegrass
KDMY	Potassium content in dry matter grain yield of maize
LEC	Land equivalent coefficient
LMERs	Linear mixed-effects models
LSAV	Land saved
LUE	Land-use efficiency
N	Nitrogen
NDMY	Nitrogen content in dry matter grain yield of maize
NMS	Nitrogen content in maize stover;
KMS	Potassium content in maize stover
PDMY	Phosphorus content in dry matter grain yield of maize
PMS	Phosphorus content in maize stover
PYD	Percentage yield difference
RCC	Relative crowding coefficient
RYT	Relative yield total
SPI	System productivity index
TKC	Total potassium content in the whole aboveground dry matter of maize
TNC	Total nitrogen content in the whole aboveground dry matter of maize
TPC	Total phosphorus content in the whole aboveground dry matter of maize
WCCs	Winter catch crops
